# Donor polymer design enables efficient non-fullerene organic solar cells

**DOI:** 10.1038/ncomms13094

**Published:** 2016-10-26

**Authors:** Zhengke Li, Kui Jiang, Guofang Yang, Joshua Yuk Lin Lai, Tingxuan Ma, Jingbo Zhao, Wei Ma, He Yan

**Affiliations:** 1 Department of Chemistry, The Hong Kong University of Science and Technology, Clear Water Bay, Kowloon, Hong Kong; 2 HKUST-Shenzhen Research Institute, No. 9 Yuexing 1st Road, Hi-tech Park, Nanshan, Shenzhen 518057, China; 3 State Key Laboratory for Mechanical Behavior of Materials, Xi'an Jiaotong University, Xi'an 710049, China; 4 Institute of Polymer Optoelectronic Materials and Devices, South China University of Technology, Guangzhou 510640, China

## Abstract

To achieve efficient organic solar cells, the design of suitable donor–acceptor couples is crucially important. State-of-the-art donor polymers used in fullerene cells may not perform well when they are combined with non-fullerene acceptors, thus new donor polymers need to be developed. Here we report non-fullerene organic solar cells with efficiencies up to 10.9%, enabled by a novel donor polymer that exhibits strong temperature-dependent aggregation but with intentionally reduced polymer crystallinity due to the introduction of a less symmetric monomer unit. Our comparative study shows that an analogue polymer with a C2 symmetric monomer unit yields highly crystalline polymer films but less efficient non-fullerene cells. Based on a monomer with a mirror symmetry, our best donor polymer exhibits reduced crystallinity, yet such a polymer matches better with small molecular acceptors. This study provides important insights to the design of donor polymers for non-fullerene organic solar cells.

## Abstract (web summary)

In organic photovoltaics, electron acceptors are developed to replace fullerenes, and new donors need to be designed to match these acceptors. Here, the authors show that a polymer with strong temperature dependent aggregation and intentionally reduced crystallinity matches non-fullerene acceptors.

## Graphical abstract

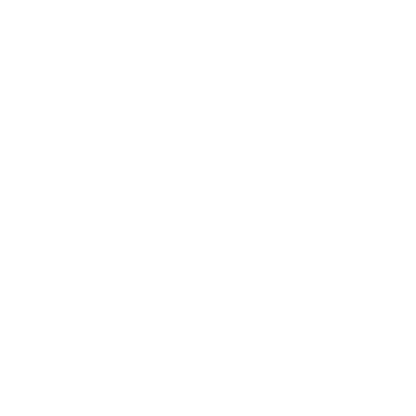

Organic solar cells (OSC) are considered a promising low-cost and environmentally friendly solar technology, as it can be produced using low-cost printing methods and does not contain any toxic components^1^,^2^,^3^,^4^,^5^,^6^. A typical OSC device consists of a pair of matching materials that function as electron donor and acceptor, respectively^6^,^7^. For the acceptor, fullerene derivatives have been the dominant choice of materials for nearly two decades and best-efficiency (over 10%) OSCs are usually achieved using fullerene acceptors^1^,^8^,^9^,^10^,^11^,^12^. However, fullerenes exhibit many drawbacks such as high-production cost and poor absorption properties^13^. To overcome these drawbacks, the OSC community has been actively exploring non-fullerene OSCs, which are believed to be the next generation of OSCs that will be more efficient and stable and cheaper than conventional fullerene devices^14^,^15^,^16^. There are several material options to construct non-fullerene OSCs. Among them, OSCs based on a polymer donor and a small molecular acceptor (SMA) have seen rapid developments in the past two years^14^,^17^. To develop efficient polymer:SMA OSCs, intensive research efforts have been devoted to the design and synthesis of novel SMA materials, which then are typically combined with known donor polymers (for example, PTB7-Th) to construct polymer:SMA OSCs (refs 16, 18, 19, 20, 21, 22, 23, 24, 25, 26, 27, 28, 29, 30, 31, 32). However, these known donor polymers were mainly designed for polymer:fullerene OSCs. Although they match well with fullerene acceptors and enable high-efficiency fullerene devices, they may not be the best matching donors for SMA materials.

To achieve efficient OSCs, the donor polymer plays a critical role in controlling the bulk-heterojunction (BHJ) morphology. One successful approach of achieving a favourable morphology (containing highly crystalline and small domains) in fullerene OSCs is the use of a family of donor polymers with strong temperature-dependent aggregation (TDA) properties, which yielded multiple cases of high-efficiency (higher than 10%) polymer:fullerene OSCs (refs 9, 12, 33). The crystallinity of these TDA polymers were much greater than conventional PTB7-family polymers, evidenced by their much larger (010) and (100) crystal size and higher hole mobility^9^,^12^. The key property is the strong TDA behavior of polymers, which leads to well-controlled aggregation of the polymer during the film cooling and drying process, resulting in highly crystalline yet small domains (20 nm) at the same time. However, it was found that the state-of-the-art TDA polymers do not perform well in SMA OSCs. For example, while PffBT4T-2OD yielded 10.8% fullerene cells, it only produced lower than 4% devices with SMAs. The successful donor polymers for fullerene cells do not appear to work best for non-fullerene OSCs and a different polymer design rationale is needed.

In this paper, we report a novel donor polymer (named PTFB-O) that enables highly efficient non-fullerene OSCs with power-conversion efficiencies (PCEs) up to 10.9%, which is near the best PCEs achievable for fullerene or non-fullerene OSCs to date. Interestingly, this donor polymer does not yield high-efficiency OSCs when combined with fullerene acceptors, yet it matches particularly well with a SMA. This shows that fullerene and SMA require very different donor polymer matches. To understand the structure–property relationship of the donor polymers and their impacts on OSC performance, we compare PTFB-O with an analogue polymer (named PTFB-P) that has a nearly identical structure, except that the position of one fluorine atom is slightly different. Surprisingly, the slight difference of fluorine position caused dramatic differences in polymer properties and their OSC performances. While PTFB-O enabled non-fullerene OSCs with >10% efficiency but fullerene OSCs with only 6.5% PCE, PTFB-P yielded non-fullerene OSCs with markedly reduced performance (7.9%) yet fullerene devices with higher efficiencies than those based on PTFB-O. The reverse trends of performance for PTFB-O and PTFB-P in fullerene or non-fullerene OSCs provide important insights into the design of donor polymers for SMA OSCs.

## Results

### Donor polymer design

**Figure 1:**
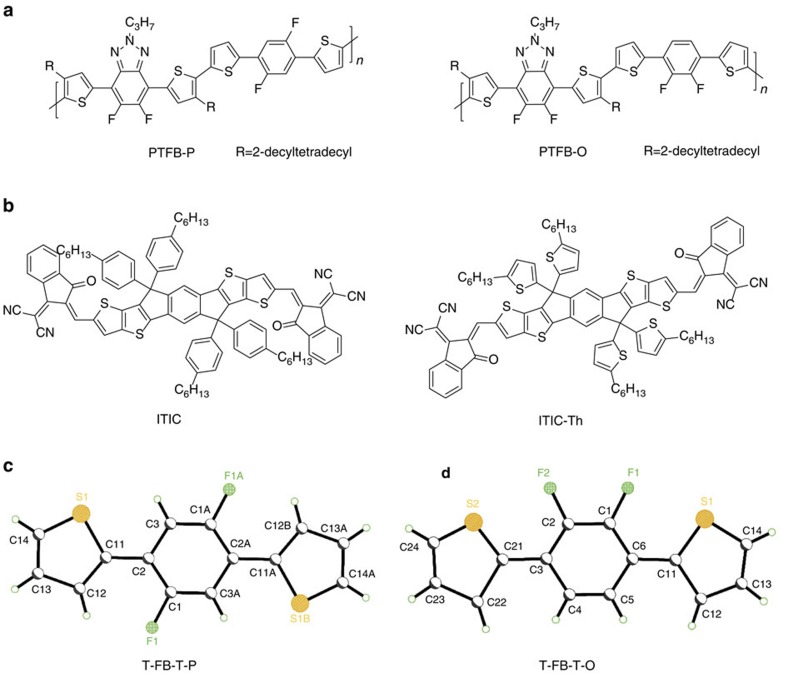
Chemical structures of polymers and SMAs. Chemical structures of (**a**) PTFB-P and PTFB-O; (**b**) ITIC and ITIC-Th. Single crystal structures of (**c**) T–FB–T-P; (**d**) T–FB–T-O (F: green, S: yellow).

The chemical structures of the two similar donor polymers are shown in Fig. 1 and the ultraviolet–visible absorption spectra are shown in Supplementary Fig. 1. These are D–A-type conjugated polymers based on an acceptor unit named difluorobenzotriazole^34^ with two flanking thiophene rings. For the donor unit, it consists of a benzene or difluorinated benzene unit inserted between two thiophene rings. For the ease of discussion, we refer to the fluorinated donor unit as T–FB–T, respectively (‘T' and ‘FB' stand for thiophene and difluorinated benzene, respectively). First, it is beneficial that the benzene unit between the two thiophenes is fluorinated, as it is well known that fluorination can promote the co-planarity of the polymer backbone and thus enhance the π–π stacking and charge transport ability of the polymer in the solid state^35^,^36^,^37^,^38^. It is important to note that the difluorinated monomer unit (T–FB–T) have two isomers with the two fluorine atoms at *para* or *ortho* positions. The single crystal structures of the two isomers are compared in Fig. 1c,d (single-crystal data summarized in Supplementary Table 1). When the two fluorine atoms are on the *para* positions of the benzene ring, the orientation of the thiophene rings are opposite to each other. For the isomer with two fluorine atoms are on *ortho* positions, the two thiophene units point to the same side where the two fluorine atoms are located. It is clear that the positions of the fluorine atoms dictate the relative orientation of the thiophene units. Similar observations of F–S interactions determining the conformation or geometry of neighbouring aromatic units have been reported before^39^. These two isomers are referred to as T–FB–T-P or T–FB–T-O respectively and the two polymers are named as PTFB-P and PTFB-O. Although there is only a minor difference in the fluorination position between the two monomers, the T–FB–T-P monomer has a C2 symmetry but the T–FB–T-O monomer exhibits a mirror symmetry, which will influence the polymer properties as revealed in the following.

### OSCs device performance

**Figure 2:**
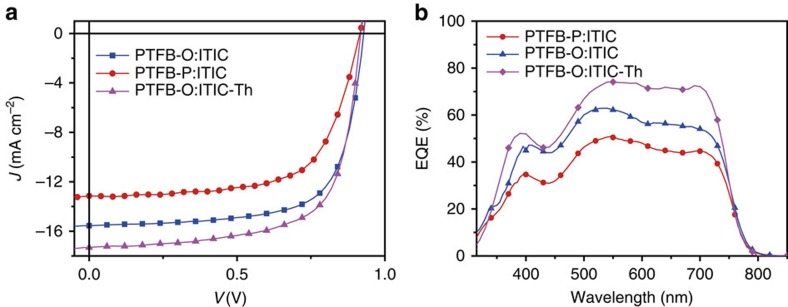
The solar cell characterization of BHJ devices prepared from polymer:SMA. (**a**) Current−voltage plots under illumination with AM 1.5G solar simulated light at 100 mW cm^−2^. (**b**) EQE spectra of the BHJ solar cells with SMA.

The performance of OSCs based on PTFB-O and PTFB-P combined with a SMA (named ITIC, Fig. 1b) or fullerene acceptor are summarized in Table 1. When ITIC was combined with PTFB-O, an impressive PCE of 10.1% was obtained, while the combination of PTFB-P and ITIC only achieved a PCE of 7.9% (Table 1, Fig. 2). However, when PTFB-O and PTFB-P were combined with PC_71_BM, PTFB-P yielded much better performance than PTFB-O (Supplementary Fig. 2). It thus appears that PTFB-O is a much more superior donor polymer match for ITIC, while PTFB-P is a better donor match for fullerene acceptor. By further optimizing the small molecule, 10.9% cell can be achieved combining PTFB-O with ITIC-Th (Fig. 1), mainly due to higher *J*_sc_, originating from the stronger absorption properties of ITIC-Th (ref. 40).

### Morphology characterization

**Figure 3:**
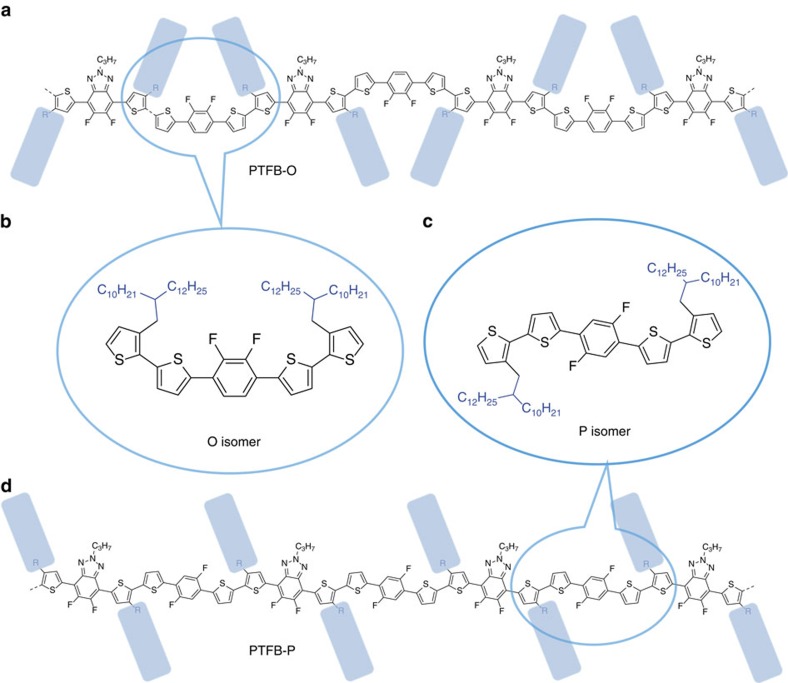
Illustration of alkyl chain orientations in polymers. (**a**) PTFB-O, (**d**) PTFB-P. Blue pane represent direction of alkyl chains. (**b**) and (**c**) Illustration of two monomer units with different symmetry (blue circle).

To understand why a subtle difference (fluorine position) in their structures causes a dramatic difference in the OSC performance, here we first investigate how the difference in fluorination position affects the polymer crystallinity. As supported by single-crystal X-ray diffraction data, the T–FB–T-P monomer has a C2 symmetry, while T–FB–T-O monomer has a mirror symmetry. The different symmetry of these two monomers dictate the relative orientation of the two alkyl chains near the T–FB–T unit. As shown in Fig. 3b (for the PTFB-O polymer), due to the mirror symmetry of the T–FB–T-O monomer, the two neighbouring alkyl chains are pointing to each other on the same side of the polymer backbone. In contrast, for the P-isomer of the PTFB-P polymer (Fig. 3c), the two alkyl chains near the T–FB–T-P unit are pointing to opposite sides of the polymer backbone, and more importantly, these two alkyl chains exhibit a parallel orientation. As a result, all the alkyl chains on the PTFB-P polymer are arranged in a parallel manner, which should facilitate the interdigitation of the alkyl chains in the solid state and promote the lamellar stacking of polymer chains.

**Figure 4:**
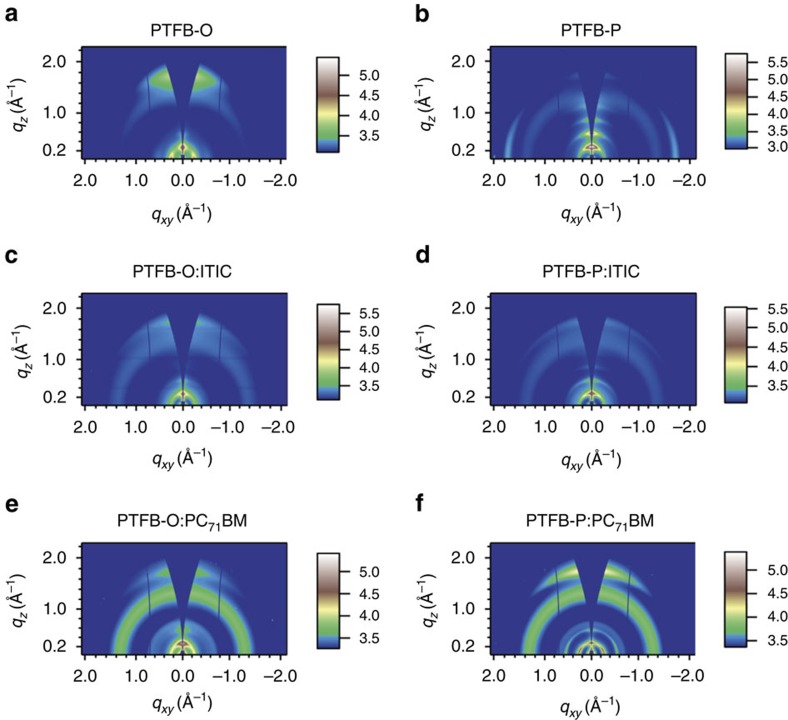
Two-dimensional GIWAXS pattern of pure polymer and polymer blend films. (**a**) PTFB-O, (**b**) PTFB-P, (**c**) PTFB-O:ITIC, (**d**) PTFB-P:ITIC, (**e**) PTFB-O:PC_71_BM and (**f**) PTFB-P:PC_71_BM. The colour scales represent the log of diffraction intensity, in the unit of counts.

To support the hypothesis above and reveal the relationship between the chemical structures and film microstructures of the two polymers, we characterize the pure PTFB-O and PTFB-P films by grazing incidence wide angle X-ray scattering (GIWAXS) and compare their polymer crystallinity. The GIWAXS two-dimensional (2D) maps of pure PTFB-O and PTFB-P films are shown in Fig. 4 and the (010) and (100) coherence length and *d* spacing data are summarized in Table 2. It is clear that PTFB-P exhibits exceptionally strong lamellar stacking as high-order diffraction peaks of (100), (200), (300) and (400) are all clearly visible. In contrast, the PTFB-O film does not exhibit high-order lamellar stacking peaks and the peak intensity is quite low. In addition, both the (010) and (100) crystal sizes of PTFB-P polymer are significantly larger than those of PTFB-O. These GIWAXS results are in good agreement with the highly regioregular structure and parallel alkyl chain arrangement of PTFB-P.

To understand the performance difference of fullerene OSCs based on PTFB-O and PTFB-P, the blend films of PTFB-O: PC_71_BM and PTFB-P:PC_71_BM were also characterized by GIWAXS. For polymer:fullerene blend films, the high polymer crystallinity of PTFB-P is maintained, as the (010) and (100) coherence length of PTFB-P:fullerene are 7.7 and 26 nm, which are significantly larger than those of PTFB-O:PC_71_BM blend. In addition, the (010) peak of PTFB-P:PC_71_BM blend changed to a preferred face-on orientation, which should be beneficial for charge transport in the vertical direction across the electrodes^41^,^42^. The hole mobilities of the blends were estimated using the space charge limited current (SCLC) method to be about 1.7 × 10^−3^ cm^2^ V^−1^ s^−1^, and 4.7 × 10^−3^ cm^2^ V^−1^ s^−1^ for PTFB-O:PC_71_BM and PTFB-P:PC_71_BM respectively (Supplementary Fig. 3). Indeed, the high polymer crystallinity of PTFB-P leads to significantly higher hole mobility, which can explain the high FF of the OSCs based on PTFB-P:PC_71_BM (refs 43, 44, 45). These results are consistent with our previous reports showing that regioregular polymers typically exhibit stronger interdigitation and thus enhanced lamellar stacking and larger crystal size^33^. These data can explain the higher FF and efficiencies of PTFB-P than PTFB-O in fullerene based OSCs.

For non-fullerene OSCs based on SMA, PTFB-O:ITIC and PTFB-P:ITIC blends were also characterized by GIWAXS and resonant soft X-ray scattering (RSoXS)^46^. Although the PTFB-P polymer is highly crystalline, GIWAXS data show that it cannot maintain its high crystallinity when blended with ITIC. As shown in Fig. 4, the scattering intensity of PTFB-P:ITIC film is low and the (010) coherence length is reduced to only 3.4 nm. Integration of the scattering intensity of the (010) peaks of PTFB-P:ITIC and PTFB-O:ITIC films show that the scattering intensity of PTFB-P:ITIC is only 50% as much as that of PTFB-O:ITIC, which indicates that there is a significantly smaller volume fraction of crystalline domain for PTFB-P:ITIC. This result is also consistent with the hole mobility data of the two blends, which showed that the PTFB-O:ITIC blend exhibits a higher SCLC mobility of 4.4 × 10^−4^ cm^2^ V^−1^ s^−1^, versus 3.3 × 10^−4^ cm^2^ V^−1^ s^−1^ for PTFB-P:ITIC.

RSoXS data revealed that the average domain size of PTFB-P:ITIC is about 50 nm, which is significantly larger than that of PTFB-O:ITIC (as shown in Supplementary Fig. 4, Supplementary Table 2). This result is also consistent with transmission electron microscopy and atomic force microscopy (AFM) images of the blend films (Supplementary Figs 4 and 5), indicative of a significantly larger domain size for PTFB-P:ITIC. Considering that the commonly accepted optimal domain size for OSCs is about 20–30 nm, the excessively large domain size of the PTFB-P:ITIC should be one of the reasons that hurts the performance of PTFB-P:ITIC-based devices. Photoluminescence (PL) quenching experiments show that the PL quenching efficiency of the PTFB-P:ITIC blend is only 75.2% at wavelength 769 nm (Supplementary Fig. 6). This indicates the exciton dissociation in the PTFB-P:ITIC blend is not as efficient as that in PTFB-O:ITIC, a result that is consistent with the larger domain size of PTFB-P:ITIC. The high PL quenching efficiency (96.0%) of the PTFB-O:ITIC blend is indicative of the absence of any excessive large, pure domains in this blend, which is a prerequisite to achieve high OSC performance. The larger domain size of PTFB-P:PC_71_BM could be due to the stronger π–π and lamellar stacking tendency of the PTFB-P polymer, which tend to stack into larger domains.

These morphology data can explain the worse performance of the PTFB-P:ITIC than PTFB-O:ITIC. The lower *J*_sc_ is likely due to the larger domain size of PTFB-P:ITIC blend, resulting in less efficient PL quenching. The lower FF of the PTFB-P:ITIC cell is due to the lower charge mobility of the PTFB-P:ITIC blend. Overall, our comparison between PTFB-O and PTFB-P show that fullerene and SMA require different donor polymers to be the best match.

## Discussion

We note that both PTFB-O and PTFB-P belong to the same polymer family as PffBT4T-2OD, as they exhibit strong TDA properties (Supplementary Fig. 7) similar to those observed for PffBT4T-2OD. Comparing their properties in details, PTFB-P exhibits a parallel arrangement of alkyl chains and thus strong lamellar stacking, and comparable crystallinity to PffBT4T-2OD. For PTFB-O, however, due to the introduction of the less symmetric monomer unit (T–FB–T-O), the parallel arrangement of alkyl chains cannot be obtained. As a result, the PTFB-O polymer exhibits weaker lamellar stacking and lower crystallinity compared with the PTFB-P and PffBT4T-2OD. Although the crystallinity of PTFB-O is relatively low within the TDA polymer family, its crystallinity and hole mobility are still significantly higher than the conventional PTB7 polymer due to its strong TDA property. The (010) coherence length of PTFB-O is 3.7 nm, which is much larger than that of PTB7, 2 nm (ref. 9). The hole mobility of PTFB-O-based blends are also about 10 × higher than those of PTB7-Th-based blends (in Supplementary Table 3). As a TDA polymer, PTFB-O is more similar to PTFB-P and PffBT4T-2OD (as they all exhibit the TDA property) than to the PTB7 polymer family. The TDA property is the key feature that allows for the achievement of an OSC morphology with good crystallinity and small domains. The difference between fullerene and SMA OSCs is that the former requires TDA polymers with strong lamellar stacking and high crystallinity (for example, PTFB-P and PffBT4T-2OD), while the latter prefers TDA polymers with slightly lower polymer crystallinity (for example, PTFB-O). Therefore, we can define PTFB-O as a new sub-class of TDA polymers whose crystallinity and lamellar stacking were reduced (compared with PffBT4T-2OD) due to the intentional introduction of less symmetric monomer units. This less crystalline sub-type of TDA polymers were found to perform better for SMA OSCs.

Similar trends of lower-crystallinity TDA polymer matching better with SMAs were also observed for the state-of-the-art PffBT4T-2OD polymer family. For example, we show (Supplementary Fig. 8) three polymers with TDA properties. Among them, PffBT4T-2OD has the strongest lamellar stacking and crystallinity due to its symmetric quaterthiophene repeating unit (GIWAXS data of PffBT4T-2OD can be found in previous report^9^). When the repeating unit of the polymer was changed to terphiophene, a polymer named PffBT-T3(1,2)-2 was obtained, which exhibits a less extent of lamellar stacking and thus lower crystallinity. When the side chains on PffBT-T3(1,2)-2 were partially (50%) replaced with longer alkyl ester chains (from C_6_C_10_ to COO-C_8_C_12_), the alkyl chain arrangement becomes even less regular,. While these polymers exhibits less crystallinity (from PffBT4T-2OD to PffBT-T3(1,2)-2 and to P3TEA)^9^,^33^,^47^, their performance in non-fullerene OSCs showed a dramatic improvement (Supplementary Table 4), because, among other reasons, the TDA polymers with intentionally reduced crystallinity can maintain a better balance between crystallinity and small domain size.

Another approach to reduce the crystallinity of the TDA polymers is to increase the size of the alkyl chains on the polymer. For example, when PffBT4T-2OD was combined with a commonly used SMA (SF-PDI_2_), a poor efficiency (3.2%) was obtained (Supplementary Table 5, Supplementary Fig. 9). Interestingly, by slightly increasing length of the alkyl chains (thus reducing polymer crystallinity) of PffBT4T-2OD, a modified polymer (PffBT4T-2DT) yielded significantly improved performance (6.3%) in non-fullerene OSCs (ref. 48). While PffBT4T-2OD and PffBT4T-2DT yielded fullerene devices with 10.9% and 7.9% efficiencies, respectively, their non-fullerene devices have a reversed trend with 3.2 and 6.3% efficiencies. PffBT4T-2DT is less crystalline and yields lower hole mobility than PffBT4T-2OD, yet it performs better with SMAs (refs 9, 48). Another similar example was also observed for a triazole-based polymer family, in which the polymer with longer alkyl chains matches better with non-fullerene acceptors^49^,^50^.

The underlying reasons for these trends may be understood as following. The advantage of the more crystalline polymer is its higher hole mobility, but the disadvantage is that it forms bigger domains for polymer:SMA blends. While high hole mobility (higher than 5.0 × 10^−3^ cm^2^ V^−1^ s^−1^) is proved to be a critical factor to achieve high FF and efficiencies for thick-film fullerene OSCs, such high mobilities may not be necessary for polymer:SMA OSCs, in which the electron mobility of SMAs (typically 10^−4^–10^−3^ cm^2^ V^−1^ s^−1^, much lower than that of fullerene) is the limiting factor. The advantage of the less crystalline polymer appears to be the smaller domain size of the polymer:SMA blends, which leads to highly efficient exciton dissociation as supported by the PL quenching data of PTFB-O:ITIC. In addition, the more crystalline polymer (PTFB-P) cannot maintain its crystallinity when blended with SMAs, the reasons of which are still under investigation.

Lastly, the high performance of our PTFB-O-based non-fullerene OSCs can be achieved without using any additives. For conventional fullerene OSCs, one approach to balance between crystallinity and small domains is using additives that were shown to reduce fullerene domain size^51^,^52^ and increase polymer crystallinity^53^,^54^,^55^ of the OSCs. These additives work well because they are bad solvents for the polymer but good solvents for the fullerene, therefore, they increase polymer crystallinity while reducing fullerene domain size. As SMAs have different solubility properties from fullerenes, it might be challenging to apply a universal additive approach to various SMAs. In fact, many SMA-based OSCs do not benefit from the use of additives^26^,^29^,^30^,^48^. For our PTFB-O-based non-fullerene OSCs, the morphology was mainly controlled by the TDA property of the polymer, therefore, the use of additives is not necessary, which is an important advantage for industry production of OSCs.

To summarize, we report a novel TDA polymer (PTFB-O) with intentionally reduced lamellar stacking and crystallinity via the introduction of a less symmetric monomer unit. While conventional TDA polymers (PffBT4T-2OD or PTFB-P) perform better when combined with fullerenes, this new type of TDA polymers were shown to match particularly well with SMAs yielding a high PCE of 10.9%. To understand why PTFB-O works well with SMAs, we compare PTFB-O with an analogue polymer (PTFB-P) with nearly identical chemical structures except for a minor difference in the fluorination position and thus the symmetry of the corresponding monomers. We show that PTFB-O and PTFB-P yielded complete opposite trends of OSC performance when they are combined with fullerene or SMA acceptors. While PTFB-O yields over 10% non-fullerene OSCs but only 6.5% fullerene cells, PTFB-P is found to be a much poor match for SMAs yet a better-performing donor polymer for fullerene OSCs. These reverse device trends can be understood by comparing the symmetry of the monomer and the crystallinity of the polymers. As the T–FB–T-P unit of the PTFB-P polymer is C2 symmetric, the alkyl chains of PTFB-P are arranged in a parallel manner, which facilitate the interdigitation of alkyl chains in the solid state and enhance the lamellar stacking and crystallinity of the polymer. In contrast, the T–FB–T-O unit of the PTFB-O polymer has a mirror symmetry, which is the reason why the PTFB-O polymer is much less crystalline. When combined with fullerenes, the more crystalline polymer PTFB-P yielded higher FF and efficiency. In case of SMA devices, the less crystalline polymer PTFB-O yielded smaller domains and higher PL quenching, which can explain the better performance of PTFB-O-based non-fullerene devices. Interestingly, the more crystalline polymer cannot maintain its crystallinity in the polymer:SMA blends, an observation that needs further studies. Our study offers an important guideline to the design of donor polymers for non-fullerene OSCs. The structure–property relationship revealed in our work should also be applicable to organic materials in other optoelectronic applications.

## Methods

### Solar cell fabrication and testing

Pre-patterned ITO-coated glass with a sheet resistance of about 15 Ω^−2^ was used as the substrate. It was cleaned by sequential sonications in soap deionized water, deionized water, acetone and isopropanol for 30 min at each step. After ultraviolet/ozone treatment for 60 min, a ZnO electron transport layer was prepared by spin coating at 5,000 r.p.m. from a ZnO precursor solution (diethyl zinc). Active layer solutions (D/A ratio 1:1.5 by weight) were prepared in chlorobenzene. To completely dissolve the polymer, the active layer solution should be stirred on a hot plate at 100 °C for at least 3 h. Before spin coating, both the polymer solution and ITO substrate are preheated on a hot plate at about 110 °C. Active layers were spin coated from the warm polymer solution on the preheated substrate in a N_2_ glovebox at 1,500–1,800 r.p.m. to obtain thicknesses of about 100 nm. The polymer:SMA films were then annealed at 90 °C for 5 min before being transferred to the vacuum chamber of a thermal evaporator inside the same glovebox. At a vacuum level of 3 × 10^−6^ Torr, a thin layer (20 nm) of MoO_3_ or V_2_O_5_ was deposited as the anode interlayer, followed by deposition of 100 nm of Al as the top electrode. All cells were encapsulated using epoxy inside the glovebox. Device *J*–*V* characteristics was measured under AM 1.5G (100 mW cm^−2^) using a Newport solar simulator (94021A, a Xenon lamp with an AM 1.5G filter) in air at room temperature. The light intensity was calibrated using a standard Si diode as a reference cell to bring spectral mismatch to unity. *J*–*V* characteristics were recorded using a Keithley 2400 source meter unit. Typical cells have devices area of 5.9 mm^2^, which is defined by a metal mask with an aperture aligned with the device area. EQEs were characterized using a Newport EQE system equipped with a standard Si diode. Monochromatic light was generated from a Newport 300 W lamp source. Additives such as DIO and CN did not result in a better performance (Supplementary Table 6). One of our best cells was sent to an accredited solar cell calibration laboratory (Enli Technology) for certification, confirming an efficiency of 10.11±0.05%, with *V*_OC_=0.9079±0.0005 V, *I*_SC_=0.999±0.005 mA, area=0.0591±0.0002, cm^2^ , FF=65.8±1.1 (Supplementary Fig. 10).

### GIWAXS characterization

GIWAXS measurements were performed at beamline 7.3.3 at the Advanced Light Source^56^. Samples were prepared on Si substrates using blend solutions identical to those used in devices. The 10 keV X-ray beam was incident at a grazing angle of 0.13–0.17°, which maximized the scattering intensity from the samples. The scattered X-rays were detected using a Dectris Pilatus 2M photon counting detector.

### RSoXS characterization

RSoXS transmission measurements were performed at beamline 11.0.1.2 at the Advanced Light Source^57^. Samples for RSoXS measurements were prepared on a poly(sodium 4-styrenesulfonate)-modified Si substrate under the same conditions as those used for device fabrication, and then transferred by floating in water to a 1.5 mm × 1.5 mm, 100-nm-thick Si_3_N_4_ membrane supported by a 5 mm × 5 mm, 200-μm-thick Si frame (Norcada). Two-dimensional scattering patterns were collected on an in-vacuum charge-coupled device camera (Princeton Instrument PI-MTE). The sample-detector distance was calibrated from the diffraction peaks of a triblock copolymer poly(isoprene-b-styrene-b-2-vinyl pyridine), which has a known spacing of 391 Å. The beam size at the sample is ∼100 μm by 200 μm. The photon energy was selected to be 284.8 eV owing to high polymer:fullerene contrast. The median domain spacing is calculated from 2π/*q*, where *q* here corresponds to half the total scattering intensity^58^. The composition variation (or relative purity of all domains) over the length scales sampled can be extracted by integrating scattering profiles to yield the integrated sector intensities. The purer the average domains are, the higher the integrated sector intensities.

### AFM analysis

AFM measurements were performed by using a Scanning Probe MicroscopeDimension 3100 in tapping mode. All film samples were spin-cast on ITO/ZnO substrates.

### Optical characterizations

Film ultraviolet–visible absorption spectra were acquired on a Perkin Elmer Lambda 20 ultraviolet/visible Spectrophotometer. All film samples were spin-cast on ITO/ZnO substrates. Solution ultraviolet–visible absorption spectra at elevated temperatures were collected on a Perkin Elmer Lambda 950 ultraviolet/visible/NIR Spectrophotometer. The temperature of the cuvette was controlled with a Perkin Elmer PTP 6+6 Peltier System, which is supplied by a Perkin Elmer PCB 1,500 Water Peltier System. Before each measurement, the system was held for at least 10 min at the target temperature to reach thermal equilibrium. A cuvette with a stopper (Sigma Z600628) was used to avoid volatilization during the measurement.

### Hole-mobility measurements

The hole-mobilities were measured using the SCLC method, employing a device architecture of ITO/V_2_O_5_/blend film/V_2_O_5_/Al. The mobilities were obtained by taking current–voltage curves and fitting the results to a space charge limited form, where the SCLC is described by:


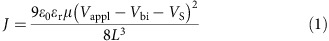


Where *ɛ*_0_ is the permittivity of free space, *ɛ*_r_ is the relative permittivity of the material (assumed to be 3), *μ* is the hole mobility, *V*_appl_ is the applied voltage, *V*_bi_ is the built-in voltage (0 V), *V*_s_ is the voltage drop from the substrate's series resistance (*V*_s_=*IR*, *R* is measured to be 10.8 Ω) and *L* is the thickness of the film. By linearly fitting *J*^1/2^ with *V*_appl_−*V*_bi_−*V*_s_, the mobilities were extracted from the slope and *L*:


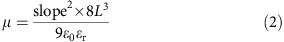


### Electron mobility measurements

The electron mobilities were measured using the SCLC method, employing a device architecture of ITO/ZnO/blend film/Ca/Al. The mobilities were obtained by taking current–voltage curves and fitting the results to a space charge limited form, where the SCLC is described by:


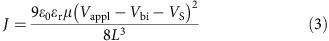


Where *ɛ*_0_ is the permittivity of free space, *ɛ*_r_ is the relative permittivity of the material (assumed to be 3), *μ* is the hole mobility and *L* is the thickness of the film. From the plots of *J*^1/2^ versus *V*, electron mobilities can be deduced. The mobilities were extracted from the slope and *L*:


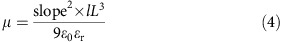


### Cyclic voltammetry

Cyclic voltammetry was performed in an electrolyte solution of 0.1 M tetrabutylammonium hexafluorophosphate, both working and counter electrodes were platinum electrode. Ag/AgCl electrode was used as the reference electrode; the Fc/Fc^+^ redox couple was used as an external standard. The energy level of PTFB-O and PTFB-P is summarized in Supplementary Fig. 11 and Supplementary Table 7.

### PL quenching measurements

PL spectra were measured on samples on ITO/ZnO substrates upon excitation of a 671-nm laser beam. The PL quenching efficiency of polymer was estimated from the ratio of the PL intensity of polymer:SMA film sample to that of the SMA control sample (Supplementary Fig. 6).

### Synthesis of PTFB-O

The detailed synthesis route can be found in Supplementary Methods. The NMR data of all the new compounds are included Supplementary Information.

### Data availability

The data that support the findings of this study are available from the corresponding author upon request.

## Additional information

**How to cite this article:** Li, Z. *et al*. Donor polymer design enables efficient non-fullerene organic solar cells. *Nat. Commun.* **7**, 13094 doi: 10.1038/ncomms13094 (2016).

## Supplementary Material

Supplementary Information Supplementary Figures 1-11, Supplementary Tables 1-7, Supplementary Methods, Supplementary References.

## Figures and Tables

**Table 1:** Photovoltaic properties of solar cells based on polymer:PC_71_BM and SMA.

**Materials**	***V***_**oc**_ **(V)**	***J***_**sc**_ **(mA cm**^**−2**^**)**	**FF**	PCE (%)*	**Best PCE (%)**
PTFB-P:PC_71_BM	0.81±0.01	12.9±0.1	0.72±0.01	7.4±0.2	7.59
PTFB-O: PC_71_BM	0.83±0.02	13.1±0.3	0.59±0.01	6.4±0.1	6.53
PTFB-P:ITIC	0.92±0.01	12.8±0.3	0.65±0.02	7.6±0.2	7.85
PTFB-O:ITIC	0.92±0.01	15.5±0.5	0.70±0.02	9.9±0.2	10.13
PTFB-O:ITIC-Th	0.92±0.01	17.1±0.5	0.67±0.02	10.5±0.3	10.88

*V*_oc_, *J*_sc_, FF and PCE represent open-circuit voltage, short-circuit current density, fill factor and power-conversion efficiency, respectively.

^*^The average values are from 30 devices.

**Table 2:** Coherence length, *d* spacing and integration of peak intensity for pure polymer, polymer:SMA and polymer:PC_71_BM films.

**Materials**	**(100) coherence length (Å)**	**(100)** ***d*** **spacing (Å)**	**(010) coherence length (Å)**	**(010)** ***d*** **spacing (Å)**
PTFB-O	115.95	24.97	37.44	3.65
PTFB-P	177.48	22.27	175.66	3.62
PTFB-O:ITIC	87.76	23.73	28.29	3.62
PTFB-P:ITIC	117.90	22.61	33.90	3.62
PTFB-O:PC_71_BM	95.59	24.54	46.44	3.62
PTFB-P:PC_71_BM	273.29	22.58	76.89	3.59
