# Supplementary material for: Donor polymer design enables efficient non-fullerene organic solar cells
Source: Nat Commun. 2016 Oct 26;7:13094. doi: 10.1038/ncomms13094 (PMC5095169; doi:10.1038/ncomms13094)
Supplement: Supplementary Information — Supplementary Figures 1-11, Supplementary Tables 1-7, Supplementary Methods, Supplementary References. [file ncomms13094-s1.pdf]

Supplementary Information

Supplementary Figures

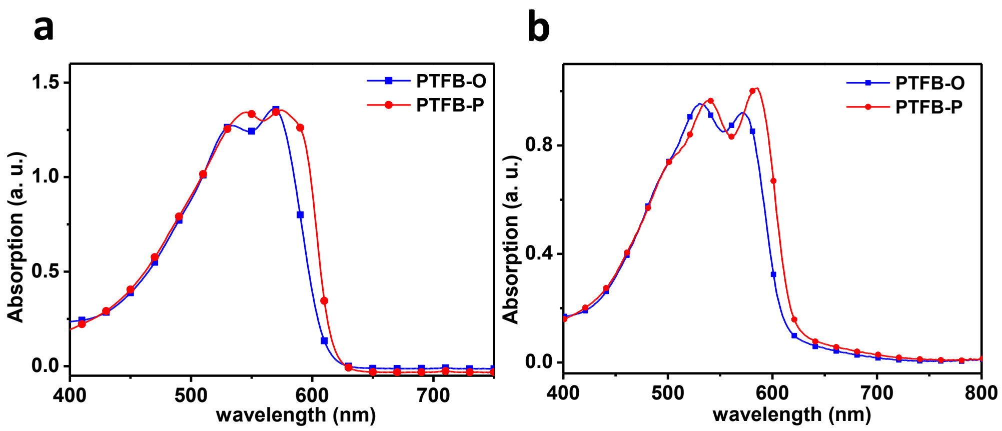

Supplementary Figure 1. Optical characterization of PTFB-O and PTFB-P**. (a)** UV-Vis absorption coefficients in solution; **(b)** comparison of the optical absorbance of pure films normalized by thickness

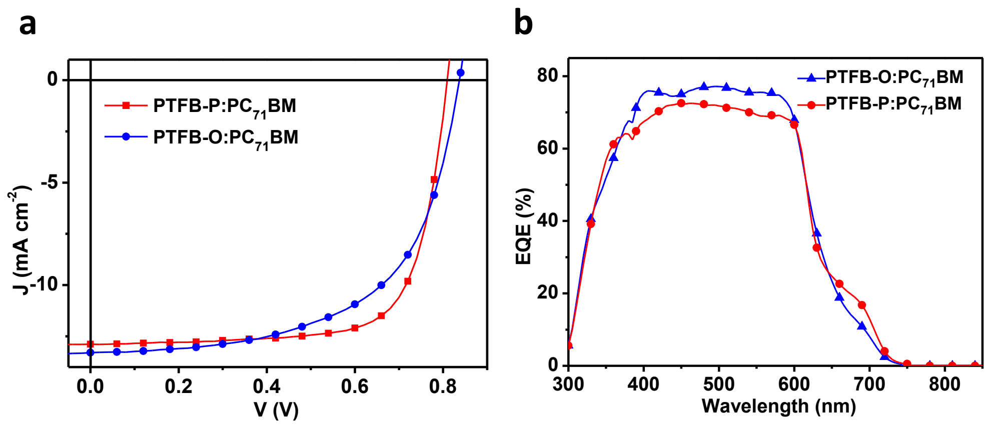

Supplementary Figure 2. Solar cell performance of polymer: PC71BM. (a) *J*–*V* curves of the solar cells. **(b)** EQE spectra of the cells.

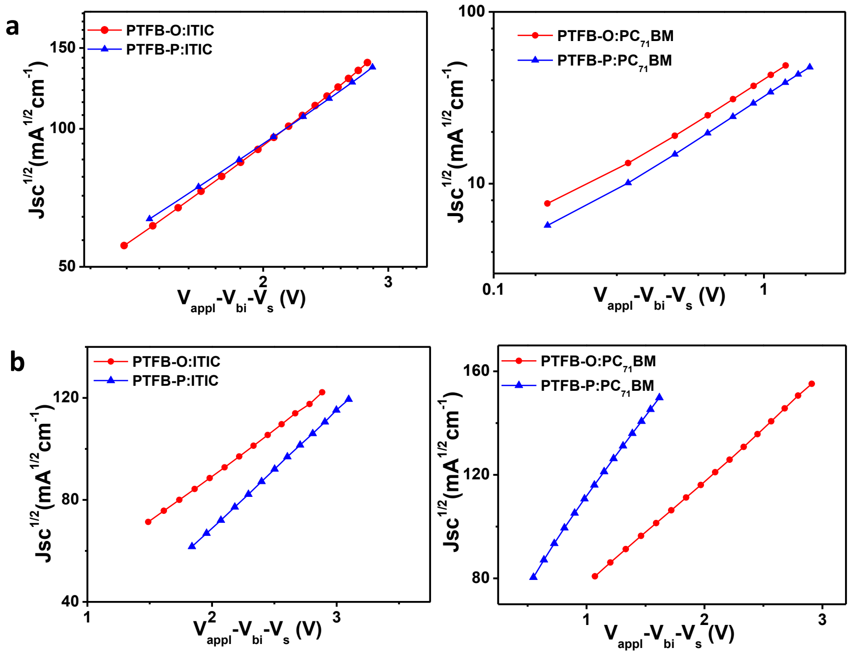

Supplementary Figure 3. $J^{1/2}$~V characteristics. (**a**) electron only and (**b**) hole-only devices of polymer:$PC_{71}BM$ and SMA, respectively.

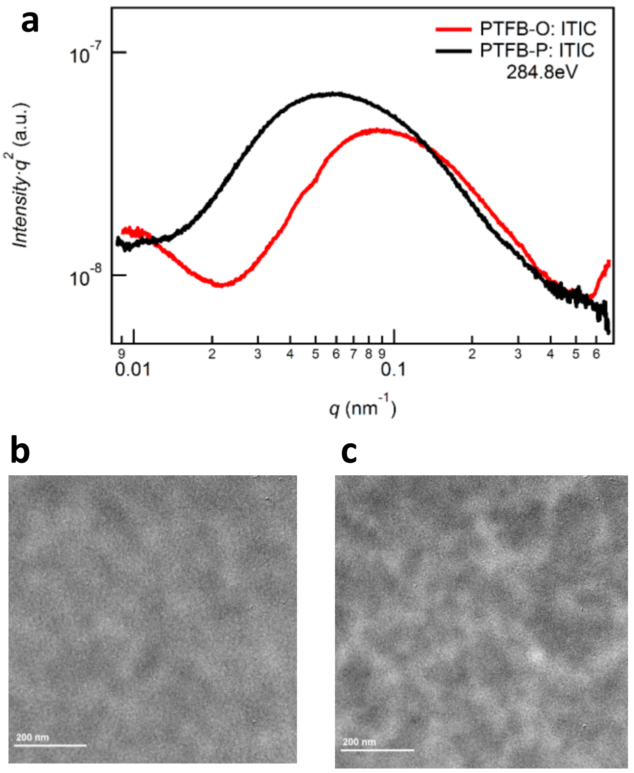

Supplementary Figure 4. R-SoXS profile and Transmission electron microscopy (200 nm) of polymer:SMA blend films. (a) R-SoXS plots of PTFB-O or PTFB-P and ITIC. TEM images of **(b)** PTFB-O:ITIC **(c)** PTFB-P:ITIC.

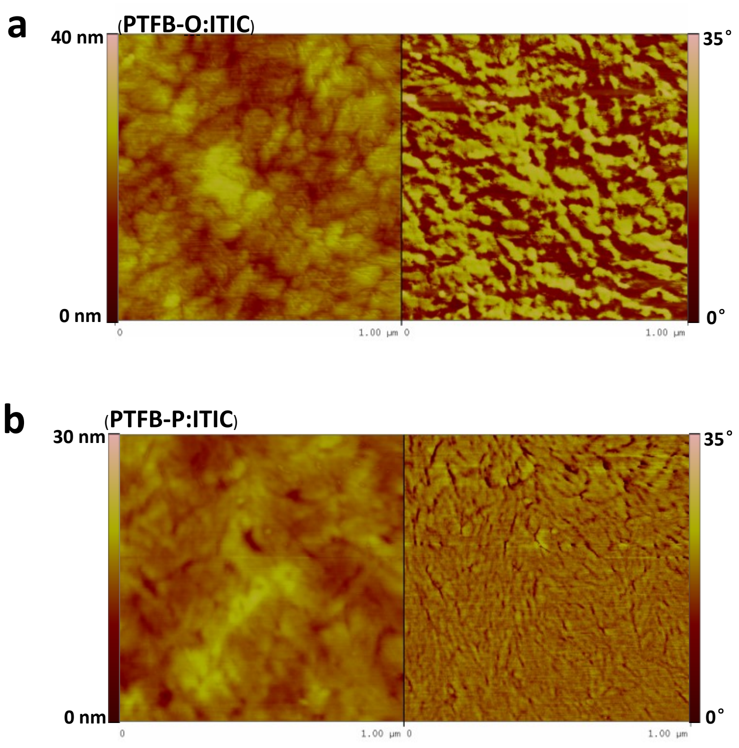

Supplementary Figure 5. **Atomic force microscopy (1×1 μm) images of polymer: ITIC**. **(a)** PTFB-O:ITIC **(b)** PTFB-P:ITIC.

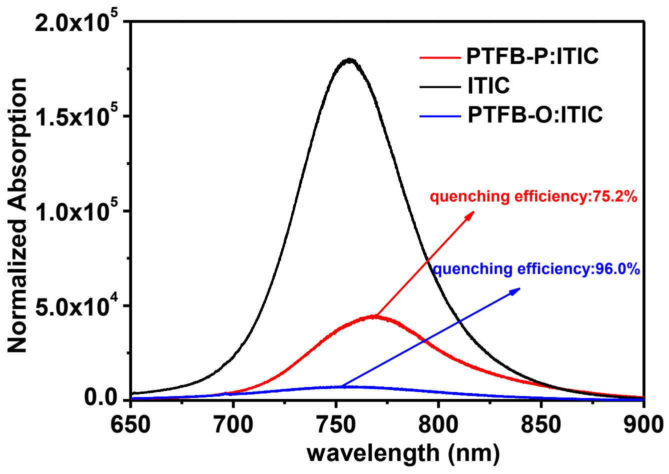

Supplementary Figure 6. Photoluminescence (PL) spectrum of PTFB-P:ITIC (red line), PTFB-O:ITIC (blue line) and ITIC (black line).

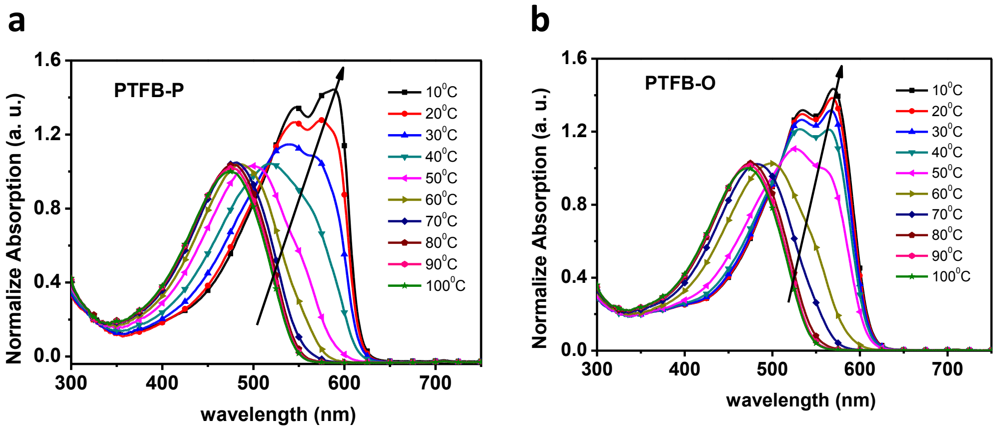

Supplementary Figure 7. UV-Vis absorption spectra evolutions of polymers. (a) PTFB-P and (b) PTFB-O in dichlorobenzene solution. (Cooling process, from 100 °C to 10 °C.)

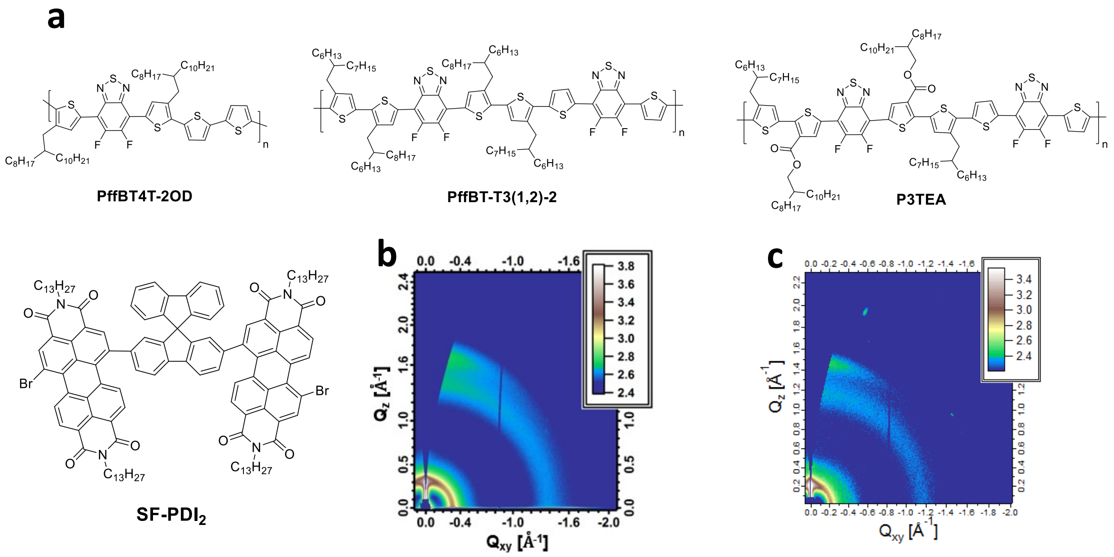

Supplementary Figure 8. Chemical structure and GIWAXS image of polymers: SMAs. (a) Chemical structure of PffBT4T-2OD, PffBT-T3(1,2)-2, P3TEA and SF-PDI$_2$; GIWAXS image of **(b)** PffBT-T3(1,2)-2 and **(c)** P3TEA.

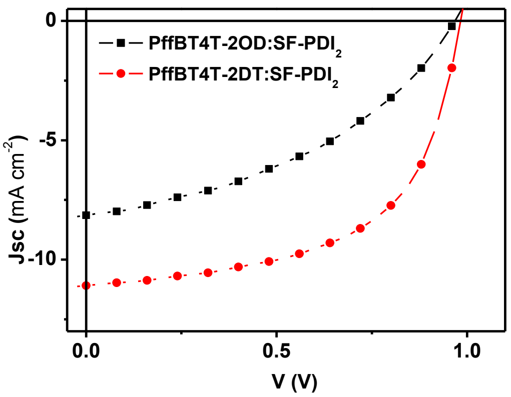

Supplementary Figure 9. Current–voltage plots under illumination with AM 1.5G solar simulated light at 100 mW cm^{-2} based on the BHJ solar cells with SMA.

Test Report

Enlitech

**Device Name:**	Photovoltaic Device
**Type:**	Non-fullerene solar cell
**Serial No.:**	PTFB-Z:ITIC-Th
**Test Date:**	2015 / 12 / 31
**Customer:**	The Hong Kong University of Science and Technology
**Address:**	The Hong Kong University of Science and Technology<br>Clear Water Bay, Kowloon, Hong Kong

The test device is measured by the laboratory and the results are given in the content.

The report consists of 4 pages including the cover and is invalid if separated.

The test results of this report are responsible to the device.

The test report should not be reproduced expect in full.

Approved by:

Hong Hsin Wu

Enli Tech. Optoelectronic Calibration Lab.

A area, 1F., No. 96, Luke 5th Rd., Kaohsiung, Taiwan, R.O.C

Enlitech Report No.: PVN160101

Device information		Environment condition	
Device Name:	Photovoltaic Device	Simulator type	Steady-State Class A
Type:	Non-fullerene solar cell	Irradiance:	1000 W/m2
Serial No.:	PTFB-Z:ITIC-Th	DUT Temperature:	25.02 ℃

Test Results and Descriptions

I. Test Results

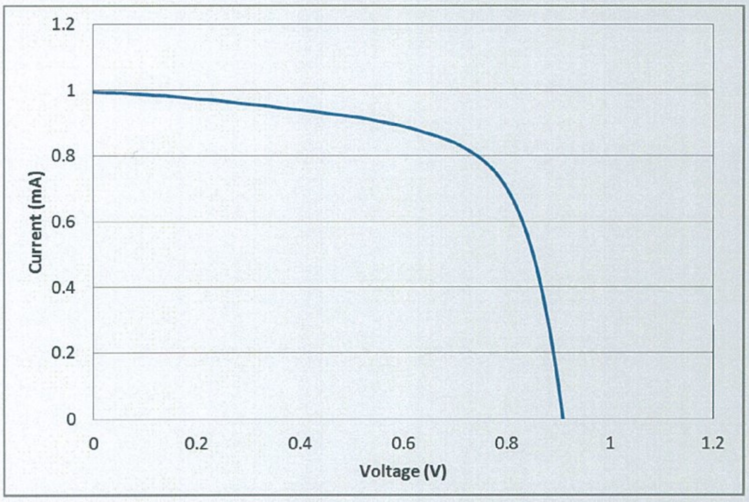

V_{OC}	=	908.10	mV	±	4.00	mV
I_{SC}	=	995.07	uA	±	13.34	uA
P_{MPP}	=	593.86	uW	±	8.32	uW

V_{MPP}	=	740.00	mV
I_{MPP}	=	802.52	uA
FF	=	65.72	%
Efficiency	=	10.07	%
Aperture area	=	0.059	cm^2

Enli Tech. Optoelectronic Calibration Lab.

A area, 1F., No. 96, Luke 5th Rd., Kaohsiung, Taiwan, R.O.C

II. Description of the test object

1 Date:

1.1 Date of Receipt: **2015 / 12 / 28**

1.2 Date of Test: **2015 / 12 / 31**

2 Test Site:

Enli Tech. Optoelectronic Calibration Lab.

3 Test Method:

The testing of the sample was performed at Standard Testing Conditions (STC) in accordance with IEC 60904-1:2006 *Photovoltaic devices - Part 1: Measurement of photovoltaic current-voltage characteristics* and *Test standard operation procedure of maximum power measurement of solar cells* [1] under the irradiation with a steady-state class AAA solar simulator according to *IEC 60904-9:2007 Photovoltaic devices - Part 9: Solar simulator performance requirements*. The spectral mismatch is calculated according to IEC 60904-7:2008 *Photovoltaic devices - Part 7: Computation of the spectral mismatch correction for measurements of photovoltaic devices*. The spectrum of the solar simulator is measured with a spectroradiometer. The spectral responsivity (or quantum efficiency) of the device under test is measured with a grating monochromatic according to IEC 60904-8:2014 *Photovoltaic devices - Part 8: Measurement of spectral responsivity of a photovoltaic (PV) device*.

4 Traceability

4-1 Traceability of Reference Cell.

Serial Number:	CGC-W1405
Organization of Calibration:	NREL
Calibration Certificate No.:	1832
Calibration Data:	2015 / 02 / 12
Traceability	NIST

4-2 The traceability of the spectral distribution to SI-Units is achieved by using a calibrated spectroradiometer.

Serial Number:	1403057U1
Organization of Calibration:	Tai Yi
Calibration Certificate No.:	K1501070401
Calibration Data:	2015 / 1 / 9
Traceability	NML

4-3 The traceability of the spectral responsivity to SI-Units is achieved by using a calibrated photo-detector.

Serial Number:	S10-15022
Organization of Calibration:	Gamma Scientific
Calibration Certificate No.:	50847-01
Calibration Data:	2015 / 9 / 17
Traceability	NIST

Enlitech Report No.: PVN160101

5 Relative Expanded Uncertainty:

5.1 Relative expanded uncertainty is estimated based on *Estimated Uncertainty Report of maximum power measurement of solar cells* [2].

5.2 The relative expanded uncertainty resulting of the relative combined standard uncertainty multiplied with a coverage factor k = 2 is specified. It corresponds to a level of confidence of 95 %.

III. Literature

1. *Test standard operation procedure of maximum power measurement of solar cells*, LAB-PV-3-1.4B, Enli Technology Co., Ltd.
2. *Estimated Uncertainty Report of maximum power measurement of solar cells*, LAB-PV-3-5.4-3B, Enli Technology Co., Ltd.

（Blank）

Enli Tech. Optoelectronic Calibration Lab.

A area, 1F., No. 96, Luke 5th Rd., Kaohsiung, Taiwan, R.O.C

Supplementary Figure 10. Solar cell performance of PTFB-O:ITIC blends certified in accredited solar cell calibration laboratory (Enli Technology).

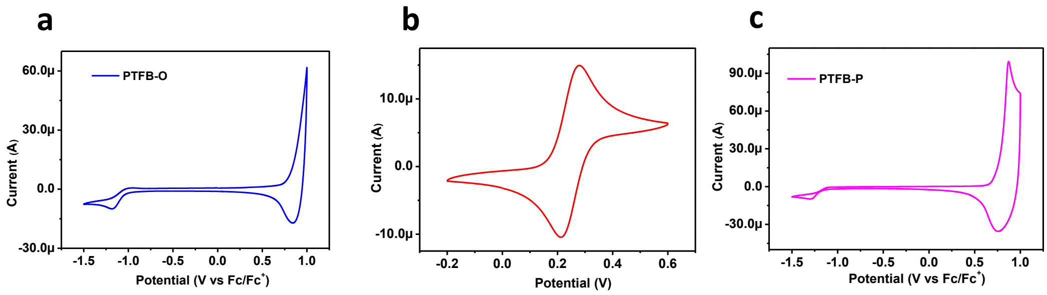

Supplementary Figure 11. Electrochemical cyclic voltammograms of polymers. (a) PTFB-O; **(b)** Ferrocene; **(c)** PTFB-P. The arrows indicate the potential onset of the oxidation or reduction reactions in the electrochemical measurements.

Supplementary Tables

Supplementary Table 1. Crystal data and structure refinement for PTFB-O and PTFB-P.

Identification code	**PTFB-O**
Empirical formula	$C_{14}H_8F_2S_2$
Formula weight	278.32
Temperature/K	100.01(10)
Crystal system	monoclinic
Space group	$P2_1$
a (Å)	6.68448(13)
b (Å)	14.7205(3)
c (Å)	11.90651(18)
α (°)	90
β(°)	97.6846(16)
γ(°)	90
Volume (Å^3)	1161.07(4)
Z	4
ρ_{calc} (g cm^{-3})	1.592
μ (mm^{-1})	4.203
F(000)	568.0
Crystal size (mm^3)	0.25 × 0.15 × 0.03
Radiation	CuKα (λ = 1.54184)
2Θ range for data collection (°)	9.606 to 133.948

Index ranges	$-6 \le h \le 7$, $-15 \le k \le 17$, $-13 \le l \le 14$
Reflections collected	6530
Independent reflections	3257 [R_{int} = 0.0171, R_{sigma} = 0.0214]
Data/restraints/parameters	3257/4/281
Completeness to theta = 66.5°	97.4%
Goodness-of-fit on F^2	1.000
Final R indexes [I>=2σ (I)]	R_1 = 0.0768, wR_2 = 0.1889
Final R indexes [all data]	R_1 = 0.0784, wR_2 = 0.1905
Largest diff. peak/hole (e Å^{-3})	1.19/-0.63

Identification code	**PTFB-P**
Empirical formula	$C_{14}H_8F_2S_2$
Formula weight	278.32
Temperature/K	100.00(10)
Crystal system	monoclinic
Space group	$P2_1/c$
a (Å)	7.51304(14)
b (Å)	3.84780(7)
c (Å)	19.8131(4)
α(°)	90
β(°)	94.3116(17)
γ(°)	90
Volume (Å^3)	571.149(19)
Z	2
ρ_{calc} (g cm^{-3})	1.618
μ (mm^{-1})	4.272
F(000)	284.0
Crystal size (mm^3)	0.22 × 0.14 × 0.12
Radiation	CuKα (λ = 1.54184)
2Θ range for data collection (°)	8.952 to 133.816
Index ranges	$-8 \le h \le 8$, $-2 \le k \le 4$, $-19 \le l \le 23$
Reflections collected	2550
Independent reflections	990 [R_{int} = 0.0093, R_{sigma} = 0.0097]
Data/restraints/parameters	990/0/88
Goodness-of-fit on F^2	1.005
Final R indexes [I>=2σ (I)]	R_1 = 0.0250, wR_2 = 0.0813
Final R indexes [all data]	R_1 = 0.0256, wR_2 = 0.0820
Largest diff. peak/hole (e Å^{-3})	0.21/-0.18

Supplementary Table 2. Summary of morphology parameters from RSoXS.

Materials	Domain Spacing (nm)	Domain purity
PTFB-O:ITIC	36.53	0.9007
PTFB-P:ITIC	50.00	0.9104
PTFB-O:$PC_{71}BM$	31.09	0.9606
PTFB-P:$PC_{71}BM$	44.03	0.9501

Supplementary Table 3. Hole and electron mobilities of polymer: $PC_{71}BM$ and SMA blends.

Materials	Hole mobility [cm^2 V^{-1} s^{-1}]	Electron mobility [cm^2 V^{-1} s^{-1}]
PTFB-O:ITIC	4.4×10^{-4}	4.30×10^{-4}
PTFB-P:ITIC	3.3×10^{-4}	3.12×10^{-4}
PTB7-Th:ITIC[1]	4.3×10^{-5}	1.1×10^{-4}
PTFB-O:$PC_{71}BM$	1.7×10^{-3}	1.60×10^{-3}
PTFB-P:$PC_{71}BM$	4.7×10^{-3}	3.50×10^{-3}
PTB7-Th:$PC_{71}BM$[2]	1.67×10^{-4}	-

Supplementary Table 4. Photovoltaic properties of the solar cells based on polymer: SMA. The average values are from over 10 devices.

Materials	V_{oc}(V)	J_{sc}(mA cm^{-2})	*FF*	*PCE* (%)
PffBT4T-2OD:SF-PDI_2	0.97	8.1	0.411	3.2
PffBT-T3(1,2)-2:SF-PDI_2	1.03	12.3	0.511	6.5
P3TEA:SF-PDI_2	1.11	13.3	0.643	9.5

Supplementary Table 5. Photovoltaic properties of the solar cells based on polymer: SMA. The average values are from over 10 devices.

Materials	V_{oc} (V)	J_{sc} (mA cm^{-2})	*FF*	*PCE* (%)	Best *PCE*
PffBT4T-2OD:SF-PDI_2	0.97±0.01	8.2±0.2	0.39±0.02	3.1±0.2	3.3
PffBT4T-2DT:SF-PDI_2	0.98±0.01	10.7±0.4	0.57±0.01	6.0±0.3	6.3
PffT2-FTAZ-2OD:IEIC	1.00±0.02	11.7±0.2	0.53±0.01	6.2±0.1	6.3
PffT2-FTAZ-2DT:IEIC	1.00±0.01	12.2±0.5	0.59±0.03	7.2±0.1	7.3

Supplementary Table 6. Photovoltaic properties of the solar cells based on PTFB-O:ITIC with DIO or CN as additives. The average values are from over 10 devices.

Materials	V_{oc} (V)	J_{sc} (mA cm^{-2})	*FF*	*PCE* (%)	Best *PCE* (%)
DIO	0.969	7.447	0.451	3.26	3.32
CN	0.870	14.17	0.652	8.03	8.26

Supplementary Table 7. HOMO Level, optical band gap and LUMO level of PTFB-O and PTFB-P.

	PTFB-O	PTFB-P
HOMO $_{CV}$ [eV] [a]	-5.36	-5.30
Bandgap [eV] [b]	2.00	1.97
LUMO $_{Opt.}$ [eV] [c]	-3.36	-3.33

[a] measured by cyclic voltammetry, [b] estimated based on film absorption onset, [c] calculated based on HOMO and optical bandgap.

Supplementary Methods

Synthetic Work: General details. All reagents and solvents were purchased from commercial sources (Aldrich, Acros, and J&K) and used without further purification. Solvents were purified by distillation when necessary. 4,7-bis(5-bromo-4-(2-decyltetradecyl)thiophen-2-yl)-5,6-difluoro-2-propyl-2H-benzo[d][1,2,3]triazole (**S4**) was synthesized according to literature procedure.[4] Microwave assisted polymerizations were conducted in a CEM Discover microwave reactor. ^{1}H and ^{13}C NMR spectra were recorded on a Bruker AV-400 MHz NMR spectrometer. Chemical shifts were reported in parts per million (ppm, δ). ^{1}H NMR and ^{13}C NMR spectra were referenced to tetramethylsilane (0 ppm) for $CDCl_3$, or solvent residual peak (5.98 ppm, ^{1}H NMR only) for $C_2D_2Cl_4$ as internal standard. Mass spectra were collected on a MALDI Micro MX mass spectrometer. Elemental analysis was performed by Midwest

Micro lab, LLC. Molecular weights of the polymers were obtained on a PL GPC 220 (Polymer Laboratories) at 135 °C using a calibration curve of polystyrene standards, with 1,2,4-trichlorobenezene as the eluent.

Materials. PffBT4T-2OD and PffBT4T-2DT were synthesized according to previous reports.[5, 6] $PC_{71}BM$ was purchased from Sigma-Aldrich. Tetrahydrofuran and toluene were freshly distilled before use from sodium using benzophenone as indicator. All other reagents and chemicals were purchased from commercial sources and used without further purification.

Synthesis procedures

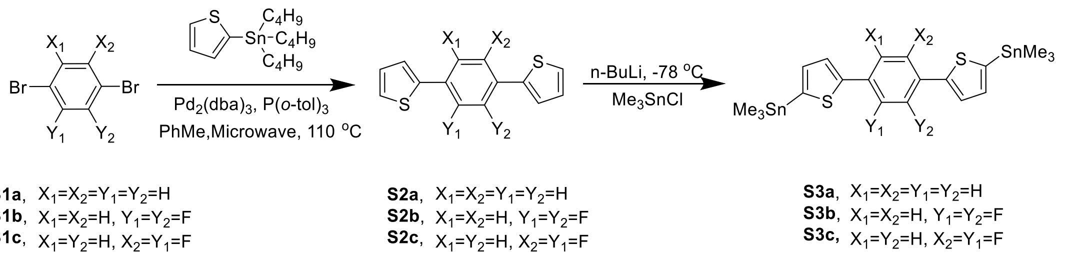

Supplementary Figure 12. Synthesis procedure of monomers.

1,4-di(thiophen-2-yl)benzene (**S2a**). To a 50 mL tube were added **S1a** (472 mg, 2.0 mmol), tributyl (thiophen-2-yl) stannane (1.87 g, 5.0 mmol), $Pd_2(dba)_3$ (91.5 mg, 0.1 mmol), P-(*o*-tol)$_3$ (182 mg, 0.6 mmol) and Toluene. The mixture was then put into microwave reactor and heated at 110 °C for 1h. After cooled to room temperature, the reaction mixture was filtered, diluted with chloroform and washed with brine 3 times. The organic layer was dried over Na_2SO_4, filtered and concentrated. Then the residue was recrystallized from isopropanol to yield pure product **S2a** as a light yellow solid (308 mg, 64% yield). ^{1}H NMR (400 MHz, $CDCl_3$) δ 7.62 (s, 4H), 7.34 (d, J = 3.6 Hz, 2H), 7.29 (d, J = 5.1 Hz, 2H), 7.11 – 7.07 (m, 2H). ^{13}C NMR (101 MHz, $CDCl_3$) δ 143.90, 133.48, 128.10, 126.29, 124.90, 123.10.
HRMS (MALDI+) Calcd for $C_{14}H_{10}S_2$ (M^+): 242.0224, Found: 242.0230.

2,2'-(2,5-difluoro-1,4-8phenylene)dithiophene (**S2b**). Synthesis of **S2b** was carried out in a similar manner to that of **S2a** using **S1b** (544 mg, 2.0 mmol), tributyl(thiophen-2-yl)stannane (1.87 g, 5.0 mmol), $Pd_2(dba)_3$ (91.5 mg, 0.1 mmol), P-(*o*-tol)$_3$ (182 mg, 0.6 mmol) and Toluene.. (406 mg, 73% yield).
^{1}H NMR (400 MHz, $CDCl_3$) δ 7.52 (d, J = 3.6 Hz, 2H), 7.40 (d, J = 5.1 Hz, 2H), 7.37 (d, J = 4.0 Hz,

2H), 7.17 – 7.11 (m, 2H). ^{19}F NMR (376 MHz, $CDCl_3$) δ -139.10 (s). ^{13}C NMR (101 MHz, $CDCl_3$) δ 148.02 (dd, *J* = 253.0, 15.9 Hz), 135.88 (s), 127.96 (s), 126.89 (t, *J* = 3.2 Hz), 126.39 (s), 122.80 (t, *J* = 3.6 Hz), 122.67 – 122.44 (m).

HRMS (MALDI+) Calcd for $C_{14}H_8F_2S_2$ (M^+): 278.0035, Found: 278.0031.

2,2'-(2,3-difluoro-1,4-phenylene)dithiophene (**S2c**). Synthesis of **S2c** was carried out in a similar manner to that of **S2b**. (420 mg, 76% yield).

^{1}H NMR (400 MHz, $CDCl_3$) δ 7.51 (d, *J* = 3.6 Hz, 2H), 7.41 (m, 4H), 7.16 – 7.11 (m, 2H). ^{19}F NMR (376 MHz, $CDCl_3$) δ -119.17 (t, *J* = 9.1 Hz). ^{13}C NMR (101 MHz, $CDCl_3$) δ 154.87 (dd, *J* = 247.5, 3.3 Hz), 135.79 (s), 127.89 (s), 126.86 (t, *J* = 3.2 Hz), 126.54 (t, *J* = 1.8 Hz), 122.25 – 121.55 (m), 115.38 (dd, *J* = 19.4, 12.1 Hz).

HRMS (MALDI+) Calcd for $C_{14}H_8F_2S_2$ (M^+): 278.0035, Found: 278.0038.

1,4-bis(5-(trimethylstannyl)thiophen-2-yl)benzene (**S3a**)

To a solution of S2a (242 mg, 1.0 mmol) in 20 mL fresh distilled anhydrous THF was added 1.6 M *n*-BuLi in hexane (1.38mL, 2.2 mmol) dropwise at -78 °C under N_2. The mixture was warmed and stirred at 0 °C for 1h. 1.0 M Me_3SnCl in hexane (2.5mL, 2.5 mmol) was then added in one portion at -78 °C and the reaction mixture was warmed to room temperature and stirred overnight. The resulted solution was then extracted by ethyl acetate 3 times. The organic layer was combined and washed with brine 3 times. The organic layer was dried over Na_2SO_4, filtered and concentrated. Then the residue was recrystallized from isopropanol to yield pure product **S3a** as a light green solid (402 mg, 71% yield).

^{1}H NMR (400 MHz, $CDCl_3$) δ 7.61 (s, 4H), 7.43 (d, *J* = 3.3 Hz, 2H), 7.17 (d, *J* = 3.4 Hz, 2H), 0.50 – 0.30 (m, 18H). ^{13}C NMR (101 MHz, $CDCl_3$) δ 149.71 (s), 137.70 (s), 136.24 (s), 133.33 (s), 126.23 (s), 124.23 (s), -8.23 (s).

HRMS (MALDI+) Calcd for $C_{20}H_{26}S_2Sn_2$ (M^+): 568.9520, Found: 568.9537.

((2,5-difluoro-1,4-phenylene)bis(thiophene-5,2-diyl))bis(trimethylstannane) (**S3b**). Synthesis of **S3b** was carried out in a similar manner to that of **S3a** using **S2b** (278 mg, 1.0 mmol), 1.6 M *n*-BuLi in hexane (1.38mL, 2.2 mmol) and Me_3SnCl (2.5mL, 2.5 mmol). **S3b** was yielded as a light yellow solid (486 mg, 81% yield).

^{1}H NMR (400 MHz, $CDCl_3$) δ 7.62 (d, J = 3.2 Hz, 2H), 7.39 (d, J = 3.9 Hz, 2H), 7.22 (d, J = 3.4 Hz, 2H), 0.50 – 0.33 (m, 18H). ^{19}F NMR (376 MHz, $CDCl_3$) δ -139.08 (d, J = 7.4 Hz). ^{13}C NMR (101 MHz, $CDCl_3$) δ 147.75 (dd, J = 252.6, 15.9 Hz), 141.59 (s), 139.52 (s), 136.02 (s), 127.83 (s), 122.84 (s), 122.44 (s), -8.20 (s).

HRMS (MALDI+) Calcd for $C_{20}H_{24}F_2S_2Sn_2$ (M^+): 605.9331, Found: 605.9329.

((2,3-difluoro-1,4-phenylene)bis(thiophene-5,2-diyl))bis(trimethylstannane) (**S3c**). Synthesis of **S3c** was carried out in a similar manner to that of **S3b**. **S3c** was yielded as a colorless solid (443 mg, 73% yield)

^{1}H NMR (400 MHz, $CDCl_3$) δ 7.60 (d, J = 3.2 Hz, 2H), 7.40 (t, J = 9.1 Hz, 2H), 7.21 (m, 2H), 0.62 – 0.27 (m, 18H). ^{19}F NMR (376 MHz, $CDCl_3$) δ -119.11 (m). ^{13}C NMR (101 MHz, $CDCl_3$) δ 154.61 (dd, J = 247.1, 3.2 Hz), 141.48 (s), 139.70 (s), 136.71 – 135.42 (m), 127.76 (t, J = 3.0 Hz), 122.21 – 121.44 (m), 115.27 (dd, J = 19.3, 12.2 Hz), -8.16 (s).

HRMS (MALDI+) Calcd for $C_{20}H_{24}F_2S_2Sn_2$ (M^+): 605.9331, Found: 605.9328.

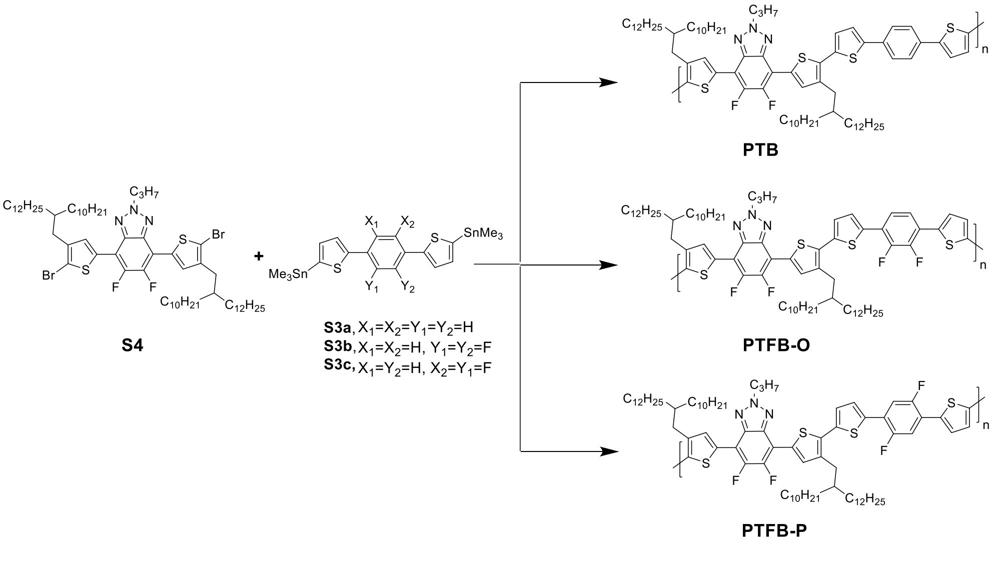

Supplementary Figure 13. Synthesis procedure of polymers.

Synthesis of PTB. To a 10 mL Microwave vial equipped with stir bar, **S4** (23.8mg, 0.02 mmol), **S3a** (11.4mg, 0.02 mmol), $Pd_2(dba)_3$ (0.3 mg) and P(o-tol)$_3$ (0.6 mg) were added. After transferred to glove

box and 0.3 mL chlorobenzene added, the vial was sealed and heated at 140 °C for 24h. Then the product was diluted with chlorobenzene and precipitated in methanol. The resulting solids were subsequently subjected to Soxhlet extraction with acetone and chloroform. After cooled to room temperature, the chloroform portion was concentrated, precipitated in methanol, collected by filtration and dried in vacuo to get the polymer as dark red solid. (16.7mg, 66 % yield):

GPC: Mn: 36.2 kDa, Mw: 71.4 kDa; PDI=1.97.

^{1}H NMR (400 MHz, $CDCl_3$) δ 8.19 (s, 2H), 7.71 (s, 4H), 7.39 (s, 2H), 7.29 (s, 2H), 4.83 (s, 2H), 2.92 (s, 4H), 2.30 (s, 2H), 1.89 (s, 2H), 1.65 – 1.05 (m, 77H), 0.91 (s, 12H).

Anal. Calcd for $C_{65}H_{93}F_4N_3S_4$: C, 74.54; H, 9.11; N, 3.30. Found: C, 74.24; H, 9.08; N, 3.20.

Synthesis of PTFB-O. To a 10 mL Microwave vial equipped with stir bar, **S4** (23.8mg, 0.02 mmol), **S3b** (12.1mg, 0.02 mmol), $Pd_2(dba)_3$ (0.3 mg) and $P(o\text{-}tol)_3$ (0.6 mg) were added. After transferred to glove box and 0.3 mL chlorobenzene added, the vial was sealed and heated at 140 °C for 24h. Then the product was diluted with chlorobenzene and precipitated in methanol. The resulting solids were subsequently subjected to Soxhlet extraction with acetone, chloroform and toluene. After cooled to room temperature, the toluene portion was concentrated, precipitated in methanol, collected by filtration and dried in vacuo to get the polymer as dark red solid. (15.0 mg, 57 % yield):

GPC: Mn: 43.8 kDa, Mw: 88.4 kDa; PDI=2.02.

^{1}H NMR (400 MHz, $CDCl_3$) δ 8.20 (s, 2H), 7.57 (s, 2H), 7.49 (s, 2H), 7.34 (s, 2H), 4.84 (s, 2H), 3.49 (s, 2H), 2.93 (s, 4H), 2.31 (s, 2H), 1.90 (s, 2H), 1.37 (d, J = 54.1 Hz, 74H), 1.18 (s, 3H), 0.92 (s, 12H).

Anal. Calcd for $C_{65}H_{93}F_4N_3S_4$: C, 72.49; H, 8.70; N, 3.21. Found: C, 72.22; H, 8.90; N, 3.20.

Synthesis of PTFB-P. To a 10 mL Microwave vial equipped with stir bar, **S4** (23.8mg, 0.02 mmol), **S3b** (12.1mg, 0.02 mmol), $Pd_2(dba)_3$ (0.3 mg) and $P(o\text{-}tol)_3$ (0.6 mg) were added. After transferred to glove box and 0.3 mL chlorobenzene added, the vial was sealed and heated at 140 °C for 24h. Then the product was diluted with chlorobenzene and precipitated in methanol. The resulting solids were subsequently subjected to Soxhlet extraction with acetone and oroform. After cooled to room temperature, the toluene portion was concentrated, precipitated in methanol, collected by filtration and dried in vacuo to get the polymer as dark red solid. (18.3 mg, 70 % yield):

GPC: Mn: 46.1 kDa, Mw: 75.8 kDa; PDI=1.64.

^1H NMR (400 MHz, $CDCl_3$) δ 8.20 (s, 2H), 7.53 (s, 4H), 7.33 (s, 2H), 4.82 (s, 2H), 2.92 (s, 4H), 2.29 (s, 2H), 1.89 (s, 2H), 1.36 (d, J = 44.3 Hz, 74H), 1.17 (s, 3H), 0.91 (s, 12H).

Anal. Calcd for $C_{65}H_{93}F_4N_3S_4$: C, 72.49; H, 8.70; N, 3.21. Found: C, 72.53; H, 8.81; N, 3.13.

Supplementary References

1 Lin, Y. et al. An Electron Acceptor Challenging Fullerenes for Efficient Polymer Solar Cells. *Adv. Mater*. **27**, 1170-1174, (2015).

2 Jiang, T. et al. Random terpolymer with a cost-effective monomer and comparable efficiency to PTB7-Th for bulk-heterojunction polymer solar cells. *Polym. Chem*. **7**, 926-932, (2016).

3 Nielsen, C. B., White, A. J. P. & McCulloch, I. Effect of Fluorination of 2,1,3-Benzothiadiazole. *J. Org. Chem.* **80**, 5045-5048, (2015).

4 Lin, H. et al. High-Performance Non-Fullerene Polymer Solar Cells Based on a Pair of Donor–Acceptor Materials with Complementary Absorption Properties. *Adv. Mater.* **27**, doi:10.1002/adma.201502775 (2015).

5 Liu, Y. et al. Aggregation and morphology control enables multiple cases of high-efficiency polymer solar cells. *Nat. Commun.* **5**, 5293, doi:Artn 5293 (2014).

6 Zhao, J. et al. High-efficiency non-fullerene organic solar cells enabled by a difluorobenzothiadiazole-based donor polymer combined with a properly matched small molecule acceptor. *Energy Environ. Sci.* **8**, 520-525, (2015).
